# Augmented monomials in terms of power sums

**DOI:** 10.1186/s40064-015-1506-5

**Published:** 2015-11-24

**Authors:** Mircea Merca

**Affiliations:** Department of Computer Science, Nicolae Grigorescu National College, Campina, 105600 PH Romania

**Keywords:** Symmetric functions, Integer partitions, 05E05, 11P81

## Abstract

The problem of base changes for the classical symmetric functions has been solved a long time ago and has been incorporated into most computer software packages for symmetric functions. In this paper, we develop a simple recursive formula for the expansion of the augmented monomial symmetric functions into power sum symmetric functions. As corollaries, we present two algorithms that can be used to expressing the augmented monomial symmetric functions in terms of the power sum symmetric functions.

## Background

Any positive integer *n* can be written as a sum of one or more positive integers, i.e.,

1 $$\begin{aligned} n=\lambda _1+\lambda _2+\cdots +\lambda _r.\end{aligned}$$

When the order of integers $\lambda _i$ does not matter, this representation is known as an integer partition Andrews (1976) and can be rewritten as

$$\begin{aligned} n=t_1+2t_2+\cdots +nt_n\end{aligned}$$

where each positive integer *i* appears $t_i$ times. If the order of integers $\lambda _i$ is important, then the representation (1) is known as a composition. For

$$\begin{aligned} \lambda _1\geqslant \lambda _2\geqslant \cdots \geqslant \lambda _r\end{aligned}$$

we have a descending composition. We notice that more often than not there appears the tendency of defining partitions as descending compositions and this is also the convention used in this paper. In order to indicate that

$$\begin{aligned} \lambda =[\lambda _1,\lambda _2,\ldots ,\lambda _r]\qquad \text {or}\qquad \lambda =[1^{t_1}2^{t_2}\ldots n^{t_n}] \end{aligned}$$

is a partition of *n*, we use the notation $\lambda \vdash n$. We denote by $l(\lambda )$ the number of parts of $\lambda$, i.e.,

$$\begin{aligned} l(\lambda )=r\qquad \text {or}\qquad l(\lambda )=t_1+t_2+\cdots +t_n. \end{aligned}$$

If $\alpha ,\beta \vdash n$, then $\alpha$ precedes $\beta$ in the dominance order if and only if for any $k\geqslant 1$, the sum of the *k* largest parts of $\alpha$ is less than the sum of the *k* largest parts of $\beta$, i.e.,

$$\begin{aligned} \alpha _1+\cdots +\alpha _k < \beta _1+\cdots +\beta _k\ \end{aligned}$$

for all $k\geqslant 1$. In this definition, partitions are extended by appending zero parts at the end as necessary. If $\alpha =[\alpha _1,\alpha _2,\ldots ,\alpha _r]$ and $\beta =[\beta _1,\beta _2,\ldots ,\beta _s]$ are partitions of the same positive integer, then $\alpha$ precedes $\beta$ in the lexicographic order if there is a positive integer *t* with the following properties:

1. $$t\leqslant r$$ and $$t\leqslant s$$;
2. for ever positive integer $i\leqslant t$, $\alpha _i=\beta _i$; and
3. either $\alpha _{t+1}<\beta _{t+1}$ or $t=r$ and $t<s$.

When $\alpha$ precedes $\beta$ in lexicographic order, we use the notation $\alpha \prec \beta$. If $\alpha \prec \beta$ or $\alpha$ = $\beta$, then we use the notation $\alpha \preceq \beta$. It is clear that the dominance order implies lexicographical order.

We recall some basic facts about monomial symmetric functions. Proofs and details can be found in Macdonald’s book (Macdonald 1995). Let $\lambda =[\lambda _1 ,\lambda _2 ,\ldots ,\lambda _k]$ be a partition with $k\leqslant n$. Being given a set of variables $\{x_1,x_2,\ldots ,x_n\}$, the monomial symmetric function

$$\begin{aligned} m_{\lambda }=m_{[\lambda _1 ,\lambda _2 ,\ldots ,\lambda _k]}(x_1,x_2,\ldots ,x_n) \end{aligned}$$

on these variables is the sum of monomial $x_1^{\lambda _1}x_2^{\lambda _2},\ldots , x_k^{\lambda _k}$ and all distinct monomials obtained from it by a permutation of variables. For instance, with $\lambda =[2,1,1]$ and $n=4$, we have:

$$\begin{aligned} m_{[2,1,1]}&=x_1^2x_2x_3+x_1x_2^2x_3+x_1x_2x_3^2+x_1^2x_2x_4\\&\quad+x_1x_2^2x_4+x_1x_2x_4^2+x_1^2x_3x_4+x_1x_3^2x_4\\&+x_1x_3x_4^2+x_2^2x_3x_4+x_2x_3^2x_4+x_2x_3x_4^2\end{aligned}$$

In particular, when $\lambda =[k]$, we have the *k*th power sum symmetric function $p_k=p_k (x_1,x_2,\dots ,x_n)$, i.e.,

$$\begin{aligned} m_{[k]}=p_k =\sum _{i=1}^{n}x_{i}^k\ . \end{aligned}$$

In every case $p_0(x_1,x_2,\ldots ,x_n)=n$.

If $\lambda \vdash k$ then $m_{\lambda }$ is a symmetric function of degree *k*. It is well-known that the set

$$\begin{aligned} \{m_{\lambda }(x_1,x_2,\ldots ,x_n)\ |\ \lambda \vdash k \ \text {and}\ l(\lambda )\leqslant n\} \end{aligned}$$

is a basis for the vector space $\Lambda _n^k$ of symmetric functions of degree *k* of *n* variables. The dimension of $\Lambda _n^k$ is the number of partitions of *k*. The power sum symmetric functions $p_k$ do not have enough elements to form a basis for $\Lambda _n^k$, there must be one function for every partition $\lambda \vdash k$. To that end in each case we form multiplicative function $p_{\lambda }=p_{\lambda }(x_1,x_2,\ldots ,x_n)$ so that for

$$\begin{aligned} \lambda =[\lambda _1,\lambda _2,\ldots ,\lambda _{l(\lambda )}]\end{aligned}$$

we note

$$\begin{aligned} p_{\lambda }=p_{\lambda _1}p_{\lambda _2}\cdots p_{\lambda _{l(\lambda)}}. \end{aligned}$$

Also, it is known that the set

$$\begin{aligned} \{p_{\lambda }(x_1,x_2,\ldots ,x_n)\ |\ \lambda \vdash k \ \text {and}\ l(\lambda )\leqslant n\} \end{aligned}$$

is another basis for $\Lambda _n^k$.

For each partition

$$\begin{aligned} \lambda =[\lambda _1,\lambda _2,\ldots ,\lambda _k]=[1^{t_1}2^{t_2}\cdots r^{t_r}]\, \end{aligned}$$

with $k\leqslant n$, the augmented monomial symmetric function

$$\begin{aligned} \tilde{m}_{\lambda }=\tilde{m}_{[\lambda _1,\lambda _2,\ldots ,\lambda _k]}(x_1,x_2,\ldots ,x_n) \end{aligned}$$

is defined by

$$\begin{aligned} \tilde{m}_{\lambda }=t_1!t_2!\cdots t_r!\cdot m_{\lambda }. \end{aligned}$$

In this paper, we develop a simple recursive formula for the expansion of the augmented monomial symmetric functions into power sum symmetric functions. As corollaries, we present two algorithms that can be used to expressing the augmented monomial symmetric functions in terms of the power sum symmetric functions.

## Two theorems for expanding augmented monomials

The cardinality of a set *A* is usually denoted $\left| A \right|$. Recall that a partition of the set *A* is a collection of non-empty, pairwise disjoint subsets of *A* whose union is *A*.

A simple way to express the augmented monomial symmetric function $\tilde{m}_{\lambda }$ in terms of the power sum is given by

### **Theorem 1**

*Let*$[\lambda _1,\lambda _2,\ldots ,\lambda _k]$*be an integer partition. Then*

$$\begin{aligned}&\tilde{m}_{[\lambda _1,\lambda _2,\ldots ,\lambda _k]}=p_{\lambda _k}\cdot \tilde{m}_{[\lambda _1,\lambda _2,\ldots ,\lambda _{k-1}]}\\&\qquad \qquad \qquad -\sum _{i=1}^{k-1}\tilde{m}_{[\lambda _1,\ldots ,\lambda _{i-1},\lambda _{i}+\lambda _{k},\lambda _{i+1},\ldots ,\lambda _{k-1}]}\, \end{aligned}$$

*where*$\tilde{m}$*andpare functions ofnvariables*, $n\geqslant k$.

### *Proof*

We denote by *M* the set of terms in the expression $p_{\lambda _k}\cdot \tilde{m}_{[\lambda _1,\lambda _2,\ldots ,\lambda _{k-1}]}$, by $M_k$ the set of terms in the expression $\tilde{m}_{[\lambda _1,\lambda _2,\ldots ,\lambda _k]}$ and by $M_i$ the set of terms in the expression $\tilde{m}_{[\lambda _1,\ldots ,\lambda _{i-1},\lambda _{i}+\lambda _{k},\lambda _{i+1},\ldots ,\lambda _{k-1}]}$, for $i=1,2,\ldots ,k-1$. According to

$$\begin{aligned} \left| M\right| =\frac{n\cdot n!}{(n-k+1)!},\quad \left| M_k\right| =\frac{n!}{(n-k)!} \end{aligned}$$

and

$$\begin{aligned} \left| M_i\right| =\frac{n!}{(n-k+1)!}, \end{aligned}$$

we get

$$\begin{aligned} \left| M\right| =\left| M_k\right| +\sum _{i=1}^{k-1}\left| M_i\right| . \end{aligned}$$

Taking into account that $\{M_i\}_{1\leqslant i \leqslant k}$ is pairwise disjoint, we deduce that $\{M_i\}_{1\leqslant i \leqslant k}$ is a set partition of *M*. Therefore, the theorem is proved. $\square$

### *Example 1*

Replacing *k* by 2 in Theorem 1, we get

2 $$\begin{aligned} \tilde{m}_{\left[\lambda_{1},\lambda_{2}\right]} = p_{\lambda_{1}}p_{\lambda_{2}}-p_{\lambda_{1}+\lambda_{2}}. \end{aligned}$$

Then, for $k=3$, we obtain

3 $$\begin{aligned} \tilde{m}_{[\lambda _1,\lambda _2,\lambda _3]}&= p_{\lambda _3}\cdot \tilde{m}_{[\lambda _1,\lambda _2]} \nonumber \\&\quad -\tilde{m}_{[\lambda _1+\lambda _3,\lambda _2]}-\tilde{m}_{[\lambda _1,\lambda _2+\lambda _3]}. \end{aligned}$$

By (2) and (3), we deduce that

$$\begin{aligned} \tilde{m}_{[\lambda _1,\lambda _2,\lambda _3]}&=p_{\lambda _1}p_{\lambda _2}p_{\lambda _3} -p_{\lambda _1}p_{\lambda _2+\lambda _3}-p_{\lambda _2}p_{\lambda _1+\lambda _3}\\&\qquad -p_{\lambda _3}p_{\lambda _1+\lambda _2}+2p_{\lambda _1+\lambda _2+\lambda _3}. \end{aligned}$$

It is clear that in the expansion of the augmented monomial $\tilde{m}_{\lambda }$ generated by Theorem 1, the number of terms is equal to the number of parts of $\lambda$.

The following result is immediate from Theorem 1.

### **Corollary 1**

*Let*$\lambda =[1^{t_1}2^{t_2}\cdots ]$*be an integer partition and letjbe a positive integer such that*$t_j>0$*. Then*

$$\begin{aligned} \tilde{m}_{\lambda }=p_j\cdot \tilde{m}_{\lambda ^0}-\sum _{i>0}(t_i-\delta _{ij})\tilde{m}_{\lambda ^i}\, \end{aligned}$$

*where*$\delta _{ij}$*is the Kronecker delta and*

$$\begin{aligned} \lambda ^i=[1^{t_1(i)}2^{t_2(i)}\cdots ]\ , \end{aligned}$$

*with*

$$\begin{aligned} t_r(0) = {\left\{ \begin{array}{ll} t_r-1, & \text {if} \quad r=j,\\ t_r, & \text {otherwise}, \end{array}\right. } \end{aligned}$$

*and*

$$\begin{aligned} t_r(i) = {\left\{ \begin{array}{ll} t_r-1-\delta _{ij}, \quad \text {if}\,r\in \{i,j\},\\ t_r+1, \quad \text {if}\,r=i+j, \\ t_r, \quad \text {otherwise} \end{array}\right. } \end{aligned}$$

*for all*$$i>0$$.

In this corollary, if $\lambda \vdash k$ then we remark that $\lambda ^0 \vdash k-j$ and $\lambda \prec \lambda ^i$ for all $i>0$ with $t_i>\delta _{ij}$. If $t_j=1$ then we have $t_j(j)=-1$. This drawback is eliminated by the fact that $t_j-\delta _{jj}=0$.

### *Example 2*

For $\lambda =[1^32^13^1]$ and $j=3$, by Corollary 1, we have

$$\begin{aligned} \lambda ^{0} = [1^32^1],\quad \lambda ^{1} = [1^22^14^1]\quad \text {and}\quad \lambda ^{2} = [1^35^1]. \end{aligned}$$

Clearly, the coefficient of $\tilde{m}_{\lambda ^0}$ is $p_3$, the coefficient of $\tilde{m}_{\lambda ^1}$ is $-3$, the coefficient of $\tilde{m}_{\lambda ^2}$ is $-1$, and for $i>2$ all the coefficients are 0. Thus, we obtain

$$\begin{aligned} \tilde{m}_{[1^32^13^1]}=p_3\cdot \tilde{m}_{[1^32^1]}-3\tilde{m}_{[1^22^14^1]}-\tilde{m}_{[1^35^1]}. \end{aligned}$$

We remark that in the expansion of $\tilde{m}_{\lambda }$ generated by Corollary 1, the number of terms is equal to

$$\begin{aligned} \text {the number of distinct parts of }\lambda + {\left\{ \begin{array}{ll} 1, \quad \text {for}\,t_j>1,\\ 0, \quad \text {for}\,t_j=1. \end{array}\right. } \end{aligned}$$

So, we can say that this corollary is a refined form of Theorem 1.

We denote by $\mathcal {P}_n$ the set of all partitions of $\left\{ 1,2,\ldots ,n \right\}$. The cardinality of the set $\mathcal {P}_n$ is well-known as the *n*th Bell number, $B_n$ (see Sloane 2012, A000110). The Möbius function of $\mathcal {P}_n$ (Bender and Goldman 1975; Rota 1964), namely

4 $$\begin{aligned} \mu (v)=\prod _{i=1}^{\left| v \right| }(-1)^{\left| v_i \right| -1}\left( \left| v_i \right| -1 \right) !, \end{aligned}$$

can be used to express the augmented monomial symmetric functions in terms of the power sum symmetric functions.

### **Theorem 2**

*Let*$\lambda$*be an integer partition. Then*

$$\begin{aligned} \tilde{m}_{\lambda }=\sum _{v \in \mathcal {P}_{l(\lambda )}}\mu (v)p_{s(v)}\, \end{aligned}$$

*where*$s(v)=[s_1,s_2,\ldots ,s_{\left| v \right| }]$*is an integer partition with*

$$\begin{aligned} s_i=\sum _{j \in v_i}\lambda _j,\quad i=1,\ldots ,\left| v \right| , \end{aligned}$$

$\tilde{m}$*andpare functions ofnvariables*, $n\geqslant l(\lambda )$.

### *Proof*

Let $\lambda =[\lambda _1,\lambda _2,\ldots ,\lambda _k]$ be an integer partition. For $v=(v_1,v_2,\ldots ,v_r)\in \mathcal {P}_{k-1}$ and $1\leqslant i \leqslant r$ let us consider $f(v),f_i(v) \in \mathcal {P}_k$ defined by

$$\begin{aligned} f(v)=(v_1,v_2,\ldots ,v_r,\{k\}) \end{aligned}$$

and

$$\begin{aligned} f_i(v)=(v_1,\ldots ,v_{i-1},v_{i}\cup \{k\},v_{i+1},\ldots ,v_r). \end{aligned}$$

By (4), we deduce that

5 $$\begin{aligned} \mu (f(v))=\mu (v)\quad \text{ and }\quad \mu (f_i(v))=-\left| v_i\right| \mu (v). \end{aligned}$$

Let $\mathcal {P'}_k$ be a subset of $\mathcal {P}_k$ defined by

6 $$\begin{aligned}{ \mathcal {P'}}_k=\left \{ v \in {\mathcal {P}}_k : \{ k \} \notin v \right \}. \end{aligned}$$

We are to prove the theorem by induction on *k*. For $k=1$, we have $\mu (\{1\})=1$ and $s(\{1\})=[\lambda _1]$. Considering that $\tilde{m}_{[\lambda _1]}=\mu (\{1\})p_{s(\{1\})}$, the base case of induction is finished. We suppose that the relation

$$\begin{aligned} \tilde{m}_{[\lambda _1,\lambda _2,\ldots ,\lambda _{k'}]}=\sum _{v \in \mathcal {P}_{k'}}\mu (v)p_{s(v)}\, \end{aligned}$$

is true for any integer $k'$, $1\leqslant k' < k$. By (5), (6) and Theorem 1, we can write

$$\begin{aligned}&\tilde{m}_{[\lambda _1,\lambda _2,\ldots ,\lambda _{k}]} \\&\quad = p_{\lambda _k}\cdot \sum _{v\in \mathcal {P}_{k-1}}\mu (v)p_{s(v)} -\sum _{i=1}^{k-1}\sum _{v\in \mathcal {P}_{k-1}}\mu (f_i(v)) p_{s(f_i(v))}\\&\quad = \sum _{v\in \mathcal {P}_{k}-\mathcal {P'}_{k}}\mu (v)p_{s(v)} +\sum _{v\in \mathcal {P'}_{k}}\mu (v) p_{s(v)}. \end{aligned}$$

Thus, the proof is finished. $\square$

### *Example 3*

For $\{1,2,3\}$, we have $\mathcal {P}_3=\{a,b,c,d,e\}$ with

$$\begin{aligned} a&= \{\{1\},\{2\},\{3\}\},\\ b&= \{\{1\},\{2,3\}\},\\ c&= \{\{2\},\{1,3\}\},\\ d&= \{\{3\},\{1,2\}\}\ \text {and}\\ e&= \{\{1,2,3\}\}. \end{aligned}$$

According to (4), we have

$$\begin{aligned} \mu (a)&=(-1)^{3-3}0!0!0!=1\,\\ \mu (b)&=\mu (c)=\mu (d)=(-1)^{3-2}0!1!=-1\ \text {and}\\ \mu (e)&=(-1)^{3-1}2!=2. \end{aligned}$$

Taking into account Theorem 2, we get

$$\begin{aligned} \tilde{m}_{[2,1,1]}=p_{[2,1,1]}-p_{[2,2]}-2p_{[3,1]}+2p_{[4]}. \end{aligned}$$

## Iterative algorithm for computing transition matrix

If $\lambda \vdash k$, then it is immediate from Theorem 1 or Theorem 2 the fact that the augmented monomial symmetric function $\tilde{m}_{\lambda }$ is a sum over integer partitions of *k*.

### **Corollary 2**

*Let*$\lambda$*be an integer partition. Then*

$$\begin{aligned} \tilde{m}_{\lambda } =\sum _{\lambda \preceq \beta }{T}_{\lambda \beta }\cdot p_{\beta } \end{aligned}$$

*where*${T}_{\lambda \beta }$*is an integer such that*

$$\begin{aligned} (-1)^{l(\lambda )-l(\beta )}{T}_{\lambda \beta }\geqslant 0, \end{aligned}$$

$\tilde{m}$*andpare functions ofnvariables*, $n\geqslant l(\lambda )$.

We observe that the transition matrix expanding the augmented monomial symmetric functions in $p_{\lambda }$ is lower triangular (with respect to any extension of the dominance ordering on partitions to a total order on the partitions $\lambda \vdash k$), i.e.,

$$\begin{aligned} \left[ \begin{array}{c} \tilde{m}_{[k^1]} \\ \vdots \\ \tilde{m}_{[1^k]} \end{array} \right] = {T}^{(k)}\cdot \left[ \begin{array}{c} p_{[k^1]} \\ \vdots \\ p_{[1^k]} \end{array} \right], \end{aligned}$$

where

$$\begin{aligned} {T}^{(k)}=\left[ {T}_{\lambda \beta } \right] _{\lambda ,\beta \vdash k}, \end{aligned}$$

with

7 $$\begin{aligned} {T}_{\lambda \beta } = {\left\{ \begin{array}{ll} 0, \quad \text {for}\, \lambda \not \preceq \beta , \\ 1, \quad \text {for}\, \lambda = \beta . \end{array}\right. } \end{aligned}$$

### *Example 4*

For $k=4$, according to Theorems 1 or 2, we obtain

$$\begin{aligned} \left[ \begin{array}{c} \tilde{m}_{[4]} \\ \tilde{m}_{[3,1]} \\ \tilde{m}_{[2^2]} \\ \tilde{m}_{[2,1^2]} \\ \tilde{m}_{[1^4]} \end{array} \right] = \left[ \begin{array}{rrrrr} 1 &{} 0 &{} 0 &{} 0 &{} 0\\ -1 &{} 1 &{} 0 &{} 0 &{} 0\\ -1 &{} 0 &{} 1 &{} 0 &{} 0\\ 2 &{} -2 &{} -1 &{} 1 &{} 0\\ -6 &{} 8 &{} 3 &{} -6 &{} 1 \end{array} \right] \left[ \begin{array}{c} p_{[4]} \\ p_{[3,1]} \\ p_{[2^2]}\\ p_{[2,1^2]}\\ p_{[1^4]} \end{array} \right]. \end{aligned}$$

We remark that

$$\begin{aligned} \tilde{m}_{[1^k]}=k!\cdot m_{[1^k]}=k!\cdot e_k, \end{aligned}$$

where $e_k$ is the *k*th elementary symmetric function. For $k=t_1+2t_2+\cdots +kt_k$, the number of ways of partitioning a set of *k* different objects into $t_i$ subsets containing *i* objects, $i=1,2,\ldots ,k$ is

$$\begin{aligned} \frac{k!}{\prod _{i=1}^{k}t_i!\cdot (i!)^{t_i}}\ \end{aligned}$$

[see (s.24.1.2 Abramovitz and Stegun 1972)]. Thus, the formula

$$\begin{aligned} {T}_{[1^k][1^{t_1}2^{t_2}\cdots k^{t_k}]}=(-1)^{k-t_1-t_2-\cdots -t_k}\frac{k!}{\prod _{i=1}^{k}t_i!i^{t_i}}, \end{aligned}$$

where $k=t_1+2t_2+\cdots +kt_k$, can be easily derived from Theorem 2. Unfortunately, for $T_{\lambda \beta }$ with $[1^k]\prec \lambda$ and $\lambda \prec \beta$ such formulas are not known.

The following result is immediate from Corollaries 1 and 2.

### **Corollary 3**

*Let kbe a positive integer. If*$\lambda =[1^{t_1}2^{t_2}\cdots ]$*and*$\beta =[1^{v_1}2^{v_2}\cdots ]$*are two integer partitions ofksuch that*$\lambda \prec \beta$*then*

$$\begin{aligned} {T}_{\lambda \beta }=\left( 1-\delta _{0,v_j} \right) {T}_{\lambda ^0\beta ^0}-\sum _{i>0}(t_i-\delta _{ij}){T}_{\lambda ^i\beta }, \end{aligned}$$

*wherejis a positive integer such that*$t_j>0$, $\delta _{ij}$*is the Kronecker delta*,

$$\begin{aligned} \beta ^0=[1^{v_1(0)}2^{v_2(0)}\cdots ]\quad \text {and}\quad \lambda ^i=[1^{t_1(i)}2^{t_2(i)}\cdots ], \end{aligned}$$

*with*

$$\begin{aligned} v_r(0)=v_r-\delta _{rj},\quad t_r(0)=t_r-\delta _{rj} \end{aligned}$$

*and*

$$\begin{aligned} t_r(i) = {\left\{ \begin{array}{ll} t_r-1-\delta _{ij}, &{} \text {if} \quad r\in \{i,j\},\\ t_r+1, &{} \text {if} \quad r=i+j, \\ t_r, &{} \text {otherwise} \end{array}\right. } \end{aligned}$$

*for all*$$i>0$$.

In this corollary, for $v_j=0$ we have $v_j(0)=-1$. Fortunately, this drawback is eliminated by the fact that $1-\delta _{0,v_j}=0$. Recall that $\lambda ^0$ is an integer partition of $k-j$ and $\lambda \prec \lambda ^i$ for all $i>0$ with $t_i>\delta _{ij}$. We remark that $\beta ^0 \vdash k-j$ for $v_j>0$.

### *Example 5*

By Corollary 3, for $\lambda =[1^4]$ and $\beta =[1^13^1]$, we have

$$\begin{aligned} T_{[1^4][1^13^1]}&=T_{[1^3][3^1]}-3T_{[1^22^1][1^13^1]}\\&=-2T_{[1^12^1][3^1]}-3\left( -2T_{[1^13^1][1^13^1]}\right) \\&=-2\left( -T_{[3^1][3^1]}\right) +6\\&=8. \end{aligned}$$

According to (7) and Corollary 3, we obtain Algorithm 1 for computing the transition matrix $T^{(k)}$. We can see that in order to compute the transition matrix $T^{(k)}$, Algorithm 1 is based on generating the immediate lexicographic predecessor of an integer partition (see lines 10 and 22). The problem of generating the immediate lexicographic predecessor of an integer partition is well-known in literature. For more details, one can refer to (Kelleher and O’Sullivan 2009) and the references therein.
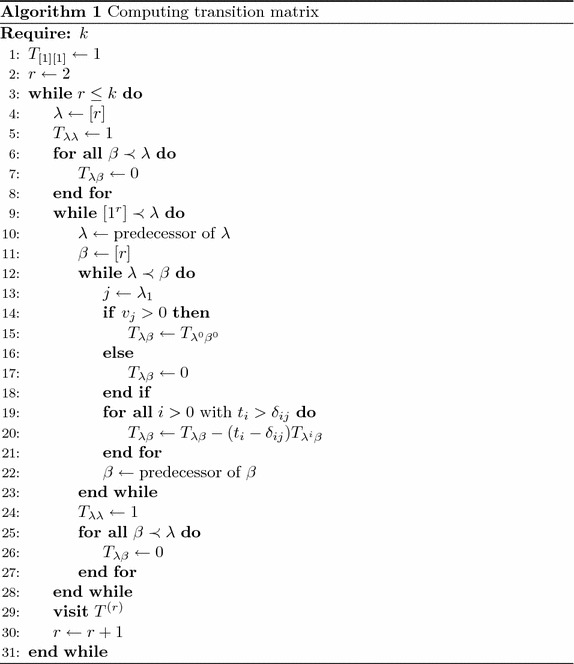


### *Example 6*

Applying Algorithm 1 for $k=5$, we get successively:

$$\begin{aligned} T^{(2)}= & {} \left[ \begin{array}{rrrrr} 1 &{} 0 \\ -1 &{} 1 \end{array} \right], \\ T^{(3)}= & {} \left[ \begin{array}{rrrrr} 1 &{} 0 &{} 0 \\ -1 &{} 1 &{} 0 \\ 2 &{} -3 &{} 1 \end{array} \right],\\ T^{(4)}= & {} \left[ \begin{array}{rrrrr} 1 &{} 0 &{} 0 &{} 0 &{} 0\\ -1 &{} 1 &{} 0 &{} 0 &{} 0\\ -1 &{} 0 &{} 1 &{} 0 &{} 0\\ 2 &{} -2 &{} -1 &{} 1 &{} 0\\ -6 &{} 8 &{} 3 &{} -6 &{} 1 \end{array} \right],\\ T^{(5)}= & {} \left[ \begin{array}{rrrrrrr} 1 &{} 0 &{} 0 &{} 0 &{} 0 &{} 0 &{} 0\\ -1 &{} 1 &{} 0 &{} 0 &{} 0 &{} 0 &{} 0\\ -1 &{} 0 &{} 1 &{} 0 &{} 0 &{} 0 &{} 0\\ 2 &{} -2 &{} -1 &{} 1 &{} 0 &{} 0 &{} 0\\ 2 &{} -1 &{} -2 &{} 0 &{} 1 &{} 0 &{} 0\\ -6 &{} 6 &{} 5 &{} -3 &{} -3 &{} 1 &{} 0\\ 24 &{}-30 &{}-20 &{} 20 &{} 15 &{} -10 &{} 1\end{array} \right]. \end{aligned}$$

At the end of this section, we remark the following

### **Conjecture 1**

*Letkbe a positive integer*. *The identities*

$$\begin{aligned} \sum _{\lambda \preceq \beta }{T}_{\lambda \beta }=0\qquad \text {and}\qquad \sum _{v \in \mathcal {P}_{l(\lambda )}}\mu (v)=0 \end{aligned}$$

*are true for all*$\lambda \prec [k]$.

## Recursive algorithm for computing an element of the transition matrix

A specific augmented monomial function $\tilde{m}_{\lambda }$ can be expressed in terms of power sums

$$\begin{aligned} \tilde{m}_{\lambda } =\sum _{\lambda \preceq \beta }{T}_{\lambda \beta }\cdot p_{\beta } \end{aligned}$$

without computing the transition matrices

$$\begin{aligned} {T}^{(r)}=\left[ {T}_{\lambda \beta } \right] _{\lambda ,\beta \vdash r},\quad r\leqslant l(\lambda ). \end{aligned}$$

According to (7) and Corollary 3, we obtain Algorithm 2 for computing the coefficient ${T}_{\lambda \beta }$ of the power sums $p_{\beta }$.

In Algorithm 2, $\lambda$ and $\beta$ are two integer partitions of *k* such that

$$\begin{aligned} \lambda =[1^{t_1}2^{t_2}\cdots k^{t_k}]\quad \text {and}\quad \beta =[1^{v_1}2^{v_2}\cdots k^{v_k}]. \end{aligned}$$

The recursive function Tlb(*k*) is presented in a form that allows fast identification of the correlation between Corollary 3 and the operations executed with the arrays $(t_1,t_2,\ldots ,t_k)$ and $(v_1,v_2,\ldots ,v_k)$. Thus, the lines 2–9 are useful to determine whether $\beta =\lambda$ or $\beta \prec \lambda$. The value of *j* is selected in the lines 16–24 such that *j* is the largest positive integer with

$$\begin{aligned} t_j=\min \{ t_i \left| t_i>0 \right. \}. \end{aligned}$$

This selection of *j* allows us to reduce the number of recursive calls from the lines 30 and 39.

The arrays $(t_1,t_2,\ldots ,t_k)$ and $(v_1,v_2,\ldots ,v_k)$ are the global variables of the recursive function Tlb(*k*). These global variables are very important because help us save memory. The integer partitions $\lambda$ and $\lambda ^i$ with $i\geqslant 0$ are alternatively stored in the same array $(t_1,t_2,\ldots ,t_k)$. The integer partition $\lambda ^0$ is immediately derived from the integer partition $\lambda$ in the line 28. Then $\lambda$ is derived from $\lambda ^0$ in the line 31. The integer partition $\lambda ^i$ with $i>0$ is derived from the integer partition $\lambda$ in the lines 36–38. Then $\lambda$ is derived from $\lambda ^i$ in the lines 40–42. The integer partitions $\beta$ and $\beta ^0$ are alternatively stored in the same array $(v_1,v_2,\ldots ,v_k)$. The integer partition $\beta ^0$ is immediately derived from the integer partition $\beta$ in the line 29. Then $\beta$ is derived from $\beta ^0$ in the line 32.

The function Tlb(*k*) can be integrated into any algorithm for generating integer partitions to get the expression of the augmented monomial $\tilde{m}_{\lambda }$ in terms of power sums.
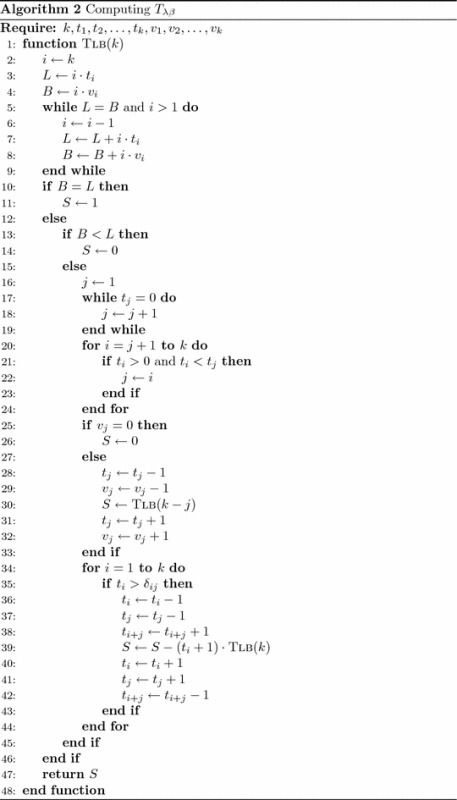


## Concluding remarks

An iterative algorithm for computing transition matrix expanding the augmented monomial symmetric functions in terms of the power sums symmetric functions has been derived in this paper. It is clear that the efficiency of this algorithm is directly influenced by the efficiency of the algorithm used for generating integer partitions in reverse lexicographic order. To express a specific augmented monomials in terms of power sums, we need a single line of the transition matrix. In this case, the computation of all transition matrix elements is not justified. Thus, a recursive function that computes the value of a single element of the transition matrix has been derived. Clearly, behind these algorithms is Theorem 1.

A recursive algorithm that requires algebraic symbol manipulation for expressing the augmented monomial $\tilde{m}_{\lambda }$ in terms of power sums can be easily derived from Theorem 1. For instance, in Maple this algorithm can be written as

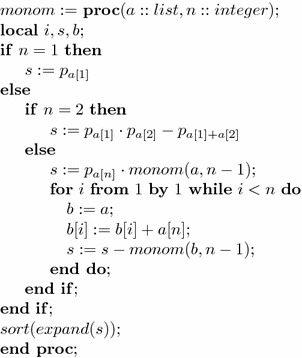



and the command

$$\begin{aligned} monom([3,2,1,1],4) \end{aligned}$$

generates the following expression

$$\begin{aligned}&p_1^2p_2p_3-p_1^2p_5-2p_1p_2p_4-2p_1p_3^2\\&\qquad -p_2^2p_3+4p_1p_6+3p_2p_5+4p_3p_4-6p_7. \end{aligned}$$

Such a recursive algorithm is very simple but its effectiveness can not be called into question because of the large number of recursive calls (for the augmented monomial $\tilde{m}_{\lambda }$ the number of recursive calls is the factorial of $l(\lambda )-1$).

Unfortunately, Theorem 2 is more difficult to exploit in order to give similar results. However, a special case can be considered.

### **Corollary 4**

*Let*$\lambda =[\lambda _1,\lambda _2,\ldots ,\lambda _r]$*be an integer partition ofksuch that*

$$\begin{aligned} \lambda _i > \lambda _{i+1}+\cdots +\lambda _r \end{aligned}$$

*for all*$$i<r$$.

1. The number of integer partition $\beta$ with $\lambda \preceq \beta$ is greater than or equal to $B_r$.
2. The number of integer partition $\beta$ with $T_{\lambda \beta }=0$ is equal to
  $$\begin{aligned} p(k)-B_r, \end{aligned}$$
  where *p*(*k*) is the Euler partition function.
3. For all $v \in \mathcal {P}_r$ the following formula holds:
  $$\begin{aligned} T_{\lambda ,s(v)}=\mu (v)\ , \end{aligned}$$
  where $s(v)=[s_1,s_2,\ldots ,s_{\left| v \right| }]$ is an integer partition with
  $$\begin{aligned} s_i=\sum _{j \in v_i}\lambda _j\ ,\quad i=1,\ldots ,\left| v \right| . \end{aligned}$$

This corollary is immediate from Theorem 2 and Corollary 3.
